# Prevalence of drug–drug interactions in geriatric patients at an ambulatory care pharmacy in a tertiary care teaching hospital

**DOI:** 10.1186/s13104-018-3342-5

**Published:** 2018-04-05

**Authors:** Rawabi Aljadani, Mohammed Aseeri

**Affiliations:** 1 Pharmacy Department, King Saud bin AbdulAziz University for Health Sciences/King AbdulAziz Medical City, Riyadh, Saudi Arabia; 2 Clinical Pharmacy Services, Pharmacy Department, King Saud bin AbdulAziz University for Health Sciences/King AbdulAziz Medical City, PO Box 9515, Jeddah, 21423 Saudi Arabia

**Keywords:** Prevalence, Drug–drug interactions, Geriatric, Ambulatory care, Pharmacy

## Abstract

**Objective:**

A cross-sectional study was performed from February to May 2015, to estimate the prevalence of drug–drug interactions in geriatric patients at the ambulatory care pharmacy at King Abdul-Aziz Medical City in Jeddah, Saudi Arabia.

**Results:**

A total of 310 patients were included, with a mean age (± SD) of 73.78 ± 6.96, and 48.70% were female. The overall prevalence of DDIs of all categories was 90.64%. Category B DDIs was 55.80%, category C DDIs 87.74%, category D DDIs 51.93%, and category X DDIs 16.45%. Atorvastatine plus omeprazole was identified as the most common interacting pair, with a prevalence of 25.26%. Multivariate logistic regression analysis showed that category D or X DDIs are more likely to occur in the female patient (OR = 1.79; 95% CI 1.07, 2.97), the patient taking more than three medications (OR = 22.62; 95% CI 2.93, 174.83), and the patient with more than two conditions (OR = 3.09; 95% CI 1.81, 5.27).

## Introduction

The issue of poly-pharmacy in geriatric patients has led to concern over an increase in drug–drug interactions (DDIs) prevalence. The precise minimum number of medications used to define “poly-pharmacy” varies but generally ranges from 5 to 10 [1]. Some combined medications may result in undesired pharmacodynamic or pharmacokinetic interactions, leading to under-treatment or harmful effects [2]. DDIs are an important cause of adverse drug reactions. Approximately 3–26% of all adverse drug reactions that require hospital admission are caused by drug–drug interactions [3]. Studies involving community-dwelling individuals report prevalence rates ranging from 4.6 to 17.6% [4, 5]. Studies conducted in hospital settings report prevalence rates ranging from 0.6 to 18.3%, and a study carried out in a primary health care unit reports a prevalence rate of 6.8% [6–8]. Janchawee studied the pharmacoepidemiologic of potential drug–drug interactions in the outpatient pharmacy of a university hospital, where the overall rate of potential DDIs was 27.9%, with a maximal value of 57.8% at the Department of Psychiatry. The rate of the most potentially significant interactions was 2.6%, being the highest in the Department of Medicine (6%), with isoniazid vs. rifampin as the most common interacting combination [9]. Another study of drug–drug interactions potential pattern in a tertiary care teaching hospital diabetic outpatient pharmacy, including a total of 182 patients, found a higher risk of DDIs among patients 51–60 years of age (43 patients, or 23.6%), It was found that 10 (5.3%) of the potential drug–drug interactions were major, 5 (2.7%) minor, and 174 (92.1%) moderate, the most common potential drug–drug interaction observed was between metformin and enalapril (n = 64) [10]. Even though nature and prevalence of potential DDIs has been widely studied in different settings, DDIs nature in the ambulatory setting still a gray area with many contradictions, ambulatory care pharmacy serves a huge number of patients on a daily basis, should provide the highest possible level of quality, with the understanding that safety assurance is a cornerstone for the quality of any type of service. We realized the harmful effect of drug–drug interactions on patients’ conditions and progress, as well as the effect on the pharmacoeconomics of the hospital itself. Based on that, this study was conducted to estimate the prevalence of drug–drug interactions in geriatric patients at the ambulatory care pharmacy at King Abdul-Aziz Medical City in Jeddah, Saudi Arabia.

## Main text

### Methods

#### Study design

This cross-sectional study was approved by King Abdullah International Medical Research Center (KAIMRC) and was conducted to estimate the prevalence of drug–drug interactions in geriatric patients at the ambulatory care pharmacy at King Abdul-Aziz Medical City in Jeddah, Saudi Arabia, from February to May 2015.

#### Inclusion and exclusion criteria

We included patients 65 years and older of both genders who visited the ambulatory care pharmacy at King Abdul-Aziz Medical City in Jeddah and who had a drug profile containing two or more medications, drug profile containing herbal products or topical products only including: creams, ointments, gels, patches, drops, sprays and inhalers were excluded.

#### Data collection

In this study, we did a retrospective review of 310 patients’ medication profiles of ambulatory care pharmacy dispensed prescriptions. The assigned pharmacist retrieved the dispensed prescriptions randomly. Then the baseline characteristic and patient demographics data (including patient age, gender, number of drugs, and chronic illness at the time of dispensing) and the patient drug profile data (including drug, dose, and route) were collected. The drug profile for each patient was analyzed by Lexi-Interact (a comprehensive drug-to-drug, drug-to-herb, and herb-to-herb interaction analysis program); Lexi-Interact categorized DDIs into five categories according to its risk rating, category A: data have not demonstrated either pharmacodynamic or pharmacokinetic interactions between the specified medications, B: the specified medications may interact with each other, but there is little to no evidence of clinical concern resulting from their concomitant use, C: the medications agents may interact with each other in a clinically significant manner, but the benefits of concomitant use of these two medications usually outweigh the risks, D: the two medications may interact with each other in a clinically significant manner, a patient-specific assessment must be conducted to determine whether the benefits of concomitant therapy outweigh the risks, and X: the specified medications may interact with each other in a clinically significant manner, but the risks associated with concomitant use of these medications usually outweigh the benefits [11].

#### Statistical analysis

Descriptive statistics were used to describe continuous (Mean ± SD) and categorical variables (frequency and percent). We used Chi square test to assess the association between D or X DDIs and categorical variables, while Independent-t test was used to assess the association between D or X DDIs and continuous variables. Prevalence was used to determine the proportion of geriatric patients visiting the ambulatory care pharmacy who have a medication profile containing at least one interacting pair with no regard for DDI risk rating. Prevalence was also used to determine the proportion of geriatric patients with at least one interacting pair for each drug–drug interaction risk rating (B, C, D, and X) alone. It was also used to estimate the proportion of patients for each one of the most common interacting pairs. Multivariate logistic regression analysis was used to determine if gender, age, number of drugs, and chronic conditions can predict D and X DDIs risk rating prevalence. The odds ratio (OR) and respective confidence interval (CI) was calculated in this analysis for each variable. Wald Chi square test P values < 0.05 were considered statistically significant. Using IBM SPSS Statistics for Microsoft, Version 22.0, we preformed all the statistical analysis [12].

### Results

A total of 310 patients were included in this study, with a mean age of 73.8 (± SD 7) years. Approximately half of them (48.70%) were female. The majority (91.3%) had a prescription with more than three medications, and 62.3% had more than two chronic conditions. Baseline characteristics and patient demographics are listed in Table 1.

**Table 1:** Baseline characteristics and patient demographics

Factor	Reference	Mean	± SD	Interactions D or X DDIs				P
				No		Yes		
				Mean	± SD	Mean	± SD	
Age		73.8	7.0	74.2	7.1	73.5	6.8	0.412
		**N**	**%**	**N**	**%**	**N**	**%**	
Gender	Female	151	48.7	56	37.1	95	62.9	0.092
	Male	159	51.3	74	46.5	85	53.5	
Number of prescribed medications	3 or fewer medications	27	8.7	26	96.3	1	3.7	0.001*
	More than 3 medications	283	91.3	104	36.7	179	63.3	
Number of chronic conditions	2 or fewer conditions	117	37.7	74	63.2	43	36.8	0.001*
	More than 2 conditions	193	62.3	56	29	137	71	

* Chi square test is significant α = 0.05

#### Drug–drug interactions prevalence

The overall prevalence of DDIs of all categories was 90.64% (95% CI 86.8%, 93.6%). Category B DDIs prevalence was 55.80% (95% CI 50.1%, 61.4%), category C DDIs 87.74% (95% CI 83.6%, 91.2%), category D DDIs 51.93% (95% CI 46.2%, 57.6%), and category X interacting pairs 16.45% (95% CI 12.5%, 21.1%).

#### Most common interacting pairs

Atorvastatin + omeprazole was identified as the most common interacting pair, with a prevalence of 25.26%. Next was atorvastatin + calcium, with a prevalence of 22.90%. The prevalence of all the identified most common interacting pairs, their risk rating, severity, reliability rating, predicted impact on the clinical outcome, and suggested patient management plan are all mentioned in Table 2.

**Table 2:** Most common interacting pairs prevalence, risk rating, severity, reliability rating, predicted impact on the clinical outcome, and patient management

Interacting pair	Prevalence (%)	Risk rating	Severity	Reliability rating	Predicted impact on the clinical outcome	Patient management
Atorvastatine + omeprazole	25.16	C	Major	Poor	Proton pump inhibitors may increase the serum concentration of HMG-CoA reductase inhibitors	Monitor for evidence of rhabdomyolysis and other adverse effects if a PPI and an HMG-CoA reductase inhibitor are co-administered
Atorvastatin + calcium	22.90	C	Minor	Fair	Antacids may decrease the serum concentration of HMG-CoA reductase inhibitors	Monitor for decreased effects of statins (e.g., cholesterol changes) in patients who consistently take antacids concomitantly
Aspirin + calcium	17.09	B	Minor	Excellent	Antacids may decrease the serum concentration of salicylates	Monitor for decreased therapeutic effects of salicylates if an antacid is initiated/dose increased or increased effects if an antacid is discontinued/dose decreased
Aspirin + metformin	16.77	C	Moderate	Fair	Salicylates may enhance the hypoglycemic effect of blood glucose lowering agents	Monitor for excessive pharmacological effect (e.g., hypoglycemia). This is likely more of a concern in patients receiving salicylates at a dose of 3 g or greater per day
Aspirin + insulin	13.87	C	Moderate	Fair		
Aspirin + gliclazide	12.25	C	Moderate	Fair		
Amlodipine + calcium	11.29	C	Moderate	Excellent	Calcium salts may diminish the therapeutic effect of calcium channel blockers	Monitor the therapeutic effects of calcium channel blockers if a calcium supplement is initiated or dose changed
Gliclazide + omeprazole	10.64	C	Moderate	Fair	CYP2C9 inhibitors (moderate) may decrease the metabolism of CYP2C9 substrates	Monitor for increased effects of the CYP substrate if a CYP inhibitor is initiated/dose increased and decreased effects if a CYP inhibitor is discontinued/dose decreased
Atorvastatin + esomeprazole	10.32	C	Major	Poor	Proton pump inhibitors may increase the serum concentration of HMG-CoA reductase inhibitors	Monitor for evidence of rhabdomyolysis and other adverse effects if a PPI and an HMG-CoA reductase inhibitor are co-administered
Atorvastatin + clopidogrel	10.32	B	Moderate	Good	Atorvastatin may diminish the antiplatelet effect of clopidogrel	No action required
Calcium + gliclazide	9.03	B	Minor Onset Rapid	Excellent	Antacids may increase the absorption of sulfonylureas. Increase in rate, not extent	Monitor for increased therapeutic effects of sulfonylureas if an antacid is administered concomitantly. Consider separating doses by at least 2 h to minimize effects
Aspirin + multivitamins/minerals	8.38	C	Moderate	Fair	Multivitamins/minerals (with AE, no iron) may enhance the antiplatelet effect of aspirin	Monitor patients closely for evidence of increased platelet inhibition (e.g., bruising, bleeding)
Alendronate + calcium	7.74	D	Moderate	Fair	Calcium salts may decrease the serum concentration of bisphosphonate derivatives	Avoid administration of oral calcium supplements 30 min after alendronate
Amlodipine + tamsulosin	7.41	C	Moderate	Excellent	Alpha1-blockers may enhance the hypotensive effect of calcium channel blockers	Monitor for increased risk of hypotension during concomitant use of an alpha1-blocker and a calcium channel blocker
Calcium + levothyroxine	7.41	D	Moderate	Fair	Calcium salts may diminish the therapeutic effect of thyroid products	Separate the doses of the thyroid product and the oral calcium supplement by at least 4 h. Monitor the therapeutic effects of thyroid products if an oral calcium supplement is initiated or dose changed
Atorvastatin + carvedilol	7.09	C	Moderate	Fair	P-glycoprotein/ABCB1 inhibitors may increase the serum concentration of P-glycoprotein/ABCB1 substrates. It may also enhance the distribution of p-glycoprotein substrates to specific tissues	Monitor the effects of P-glycoprotein (Pgp) substrates if a Pgp inhibitor is started or if the dose of a concurrently used Pgp inhibitor changed

#### Drug–drug interactions predictors

Univariate logistic regression analysis including all 310 patients showed that the odds of D or X DDIs were 44 times higher when more than three medications were prescribed (OR = 44.75, 95% CI 5.99, 334.61). The percentage of D or X DDIs was higher in patients taking more than three medications (3.7% were using ≤ 3 medications vs. 63.3% using > 3 medications, P = 0.001). Also, univariate analysis revealed that the odds of D or X DDIs were four times higher when the patient had more than two chronic conditions (OR = 4.21, 95% CI 2.59, 6.86). When we compare the number of chronic conditions among patients with D or X DDIs, we can see that the percentage of patients with more than two conditions who have D or X DDIs almost doubles the percentage of patients with two or fewer conditions (36.8% with ≤ 2 conditions vs. 71.0% > 2 conditions, P = 0.001). The combined effects of all the factors were further investigated by conducting a multivariate logistic regression analysis, which indicated that D or X DDIs were more likely to occur in the female patient (OR = 1.79; 95% CI 1.07, 2.97), the patient taking more than three medications (OR = 22.62; 95% CI 2.93, 174.83), and the patient with more than two conditions (OR = 3.09; 95% CI 1.81, 5.27). Regression analysis results are stated in Table 3.

**Table 3:** Logistic regression analysis results for category D or X DDIs potential predictors

Factor	Reference	Univariate analysis				Multivariate analysis			
		OR	95% CI for OR		P	OR	95% CI for OR		P
			Lower	Upper			Lower	Upper	
Age		0.99	0.96	1.02	0.411	0.983	0.95	1.02	0.348
Female	Male	1.48	0.94	2.33	0.092	1.79	1.07	2.97	0.026*
More than 3 medications	3 or fewer medications	44.75	5.99	334.61	0.001*	22.62	2.93	174.83	0.003*
More than 2 conditions	2 of fewer conditions	4.21	2.59	6.86	0.001*	3.09	1.81	5.27	0.001*

* Wald Chi square test is significant α = 0

### Discussion

The overall DDIs prevalence estimate (90.64%) could be misleading if not linked to the expected clinical impact of each category, to correctly judge these results, we must consider that category C, which has the highest influence on this estimate, has less of a clinical impact compared to category D and X DDIs. D and X DDIs’ estimated prevalence of 51.93 and 16.45% respectively reflects the need for in-depth investigations. Björkman et al. studied drug–drug interactions in elderly outpatients in six European countries. They found that 46% of patients had at least one potential clinically significant DDI, and 10% of these DDIs were classified as (to be avoided) according to the Swedish interaction classification system [13]. Venturini et al. reported a moderate DDIs prevalence of 69.9%, and a 21.2% prevalence of major DDIs. The difference in the results may be attributed to the use of different DDIs analysis database; DDIs categories differ according to the database used, the difference in the population characteristics, and the sample size of these studies [14]. A direct comparison can’t be done without a further adjustment for these factors. Other studies reported the prevalence of DDIs either in different settings, such as the hospital, or in a specific population, such as cancer patients, so also can’t be directly compared with the results of this study.

Several studies investigated the most common interacting pairs, and different results were reported. This is to be expected due to the variations in the medication availability and medical practice of each institute. Of the top 20 identified most common interacting pairs we identified, atorvastatine was responsible for four, as was aspirin. Hypoglycemic agents, PPIs, and calcium were each responsible for three interacting pairs. Dinesh reported aspirin as the fourth-ranked of the top 10 medications with a high risk for DDIs [10].

Our multivariate logistic regression analysis results agree with the results of many previous studies. Neto et al. studied the prevalence and predictors of potential drug–drug interactions in the elderly in the Brazilian primary public health system. Their multivariate regression analysis results showed that being female and having three or more diagnosed diseases were positively associated with exposure to DDIs (OR = 2.49; 95% CI 2.29, 2.75 and OR = 6.43; 95% CI 3.25, 12.44, respectively) [15]. Teixeira et al. performed a multivariate regression that showed that patients with three to five medications have high odds of DDIs (OR = 4.84; 95% CI 2.85, 7.91), while patients with six or more medications have higher odds of DDIs (OR = 25.11; 95% CI 9.98, 48.63) [16].

### Conclusion

The prevalence of DDIs among geriatric patients attending the ambulatory care pharmacy was high. This indicates the need for special care while handling their prescriptions. Even though the majority of the most common interacting pairs (top 20) were category C, we still need to look for D and X interacting pairs carefully. Finally, the identified DDIs’ main predictors of number of prescribed medications (more than four) and number of chronic conditions (more than three) could guide us to provide better care and to develop a safer practice.

## Limitations

DDIs analysis was based only on one database (Lexi-Interact). Our study was conducted in a specific type of setting and population, which is why our findings are not generalizable to other settings or different populations, especially populations such as cancer or transplant patients. The study may underestimate the prevalence of DDIs in these populations. Additionally, non-prescribed medications such as over-the-counter and herbal products were excluded, which may lead to an underestimate of the prevalence of DDIs in our sample. Despite these limitations, the results of this study represent a step forward in exploring the occurrence and nature of DDIs in geriatric patients attending the ambulatory care pharmacy.

## Abbreviations

DDIs: drug–drug interactions
KAIMRC: King Abdullah International Medical Research Center
